# Utilizing Public Health Frameworks and Partnerships to Ensure Equity in DNA-Based Population Screening

**DOI:** 10.3389/fgene.2022.886755

**Published:** 2022-05-13

**Authors:** Elyse Azriel, Candace Henley, Joan Ehrhardt, Heather Hampel, Anna Newlin, Erica Ramos, Catherine Wicklund, Debra Duquette

**Affiliations:** ^1^ Northwestern University, Feinberg School of Medicine, Chicago, IL, United States; ^2^ The Blue Hat Foundation, Chicago, IL, United States; ^3^ City of Hope National Medical Center, Duarte, CA, United States; ^4^ NorthShore University HealthSystem, Evanston, IL, United States; ^5^ Genome Medical, San Francisco, CA, United States

**Keywords:** genomic screening, DNA-based screening, public health, population screening, health equity

## Abstract

DNA-Based population screening in the United States has the promise to improve the health of all people in all communities. We highlight recent DNA-based population screening examples at the state, local, and individual level. Key public health principles and concepts with a focus on equity appear to be lacking in current efforts. We request ‘A Call to Action’ that involves all partners in DNA-based population screening. Potential actions to consider include: a) identification and elimination of systemic barriers that result in health inequities in DNA-based population screening and follow-up; b) creation of a national multidisciplinary advisory committee with representation from underserved communities; c) revisiting well-described public health screening principles and frameworks to guide new screening decisions and initiatives; d) inclusion of the updated Ten Essential Public Health Services with equity at the core in efforts at the local, state and national level.

## Introduction

The vision of precision public health is ‘providing the right intervention to the right population at the right time’ (Khoury et al., 2016). In order to achieve this vision, it is critical to integrate current public health principles and frameworks in the development and implementation of population-level genomic screening (Andermann et al., 2008; The Futures Initiative, 2020). These revised frameworks have placed a stronger focus on equity. It is imperative that all DNA-based population screening efforts at all levels center equity to improve the health for all people in all communities. We discuss the public health framework for decision making and implementation using the example of population-based newborn screening (NBS). We also provide recent examples of DNA-based population screening at the individual, local, and state levels to highlight the importance of equity and partnerships.

## Existing Health Inequities in Genetic Services

Health care inequity is defined as a difference in treatment provided to members of different groups that is not justified by the underlying health conditions or treatment preferences of patients (National Academies of Sciences, Engineering, and Medicine, 2018). With the introduction of any new technology into health care, there are significant concerns that all segments of the population - especially medically underserved groups—will not be reached (National Academies of Sciences, Engineering, and Medicine, 2018). This is especially true for genetic technologies and precision medicine. Access to genetic services in the United States is primarily gated by referrals from non-genetics providers for patients with a significant personal and/or family history based on clinical guidelines. This has resulted in stark inequities to genetic services with multiple barriers at the individual, provider, and healthcare system levels (Childers et al., 2018; Chapman-Davis et al., 2021; Weise et al., 2021). For example, physicians who serve a high proportion of minority patients are significantly less likely to have ever referred a patient for genetic counseling and testing (Shields et al., 2008). There is also less awareness of genetic testing among individuals who identify as Hispanic or non-Hispanic Black and live in rural areas (Salloum et al., 2018). Disparities in access to and awareness of genomic medicine is a complex issue that affects several populations, including underrepresented minorities, rural communities, medically underserved groups, and others (National Academies of Sciences, Engineering, and Medicine, 2018).

The experiences of Candace Henley, cancer survivor and Lynch syndrome patient, highlights these barriers and conveys the need for an urgent focus on equity:

“The opportunity to have been proactive to avoid my cancer diagnosis and the devastating after-effects would have been ideal. The words “you have colon cancerˮ echoed in my head and left me numb, and everything else said to me afterward was lost to thoughts about my children and what would happen if I died. I was shocked because I was 35 years old, with a disease that occurs in people over 50; how?

The first and last time Lynch Syndrome was mentioned was a brief conversation at the six-week visit after my surgery; genetic testing or referral to a counselor was never offered or suggested. Combing through medical records from my diagnosis in 2003, it simply said: “MSI associated.ˮ That was the pathology report.

For years, I thought Lynch Syndrome was something I should be proud of until I learned from other survivors and medical professionals at conferences that it was not. 11 years after my diagnosis, I learned my father and two aunts were diagnosed post-autopsy with colon cancer.

In communities of color, doctors are not recommending genetic testing at the same rate as whites are. In addition, patient barriers exist, such as access to information about and education about genetic testing, racial inequities in care, lack of trust, physician perception of barriers such as psychological distress, and unconscious or implicit bias. Knowledge leads to prevention, healthier patient outcomes, and builds trust. Everyone deserves the opportunity to fight their best fight against cancer or any other illness.”

Additionally, the disparities across state and federal insurance health insurance plans fundamentally contribute to disparities for patients. Although percentages vary by state, 86% of Medicare beneficiaries are covered due to being age 65 and older and 14% are covered due to disability across the U.S. (Kaiser Family Foundation, 2019). Medicare coverage specifically creates two gaps that exacerbate disparities. First, genetic testing is only a covered benefit if the individual has the condition of interest, and the testing will be used for clinical decision-making. As such, those who are healthy but at risk are not eligible to have testing covered by Medicare. Second, genetic counselors are not currently recognized as providers by the Centers for Medicare & Medicaid Services, so individuals with Medicare are dependent on providers with less training in genetics to offer and manage the appropriate testing. This second issue is critical to all individuals seeking genetic testing, regardless of whether they have Medicare or a commercial third-party payor. A recent study by Lin et al. (2022) assessed the barriers to genetic testing access in academic medical centers and safety net hospitals in California and North Carolina. Both types of institutions reported that the lack of coverage of genetic counseling was a “major barrier to testing”. These are important gaps that will require significant changes in payer policies to implement DNA-based population screening efforts. Currently, DNA-based population efforts are not funded by health insurers and therefore do not suffer from these same issues.

The traditional clinical guidelines referral approach has also resulted in incomplete and inaccurate information regarding genetic disease prevalence, penetrance and natural history. There are numerous recent studies that have demonstrated that DNA-based population screening efforts not only detect more individuals in the population with genetic disease, but also add to our knowledge regarding the spectrum of disease especially in disparate populations (Manickam et al., 2018; Yang et al., 2018; Buchanan et al., 2020; Grzymski et al., 2020).

## Newborn Screening Principles and Infrastructure: Lessons for DNA-Based Population Screening

DNA-based population screening efforts can benefit from the lessons learned over the past 50 years of newborn screening. For instance, newborn screening utilizes an established framework to prioritize specific conditions, a strategy that would be beneficial for DNA-based population screening programs to adopt. The gold standard in screening policy decisions, not limited to newborn screening, is the Wilson and Jungner criteria (Andermann et al., 2008). Wilson and Jungner first published their instrumental work “Principles and Practice of Screening for Disease” in 1968 (Wilson and Junger, 1968). Their focus was mainly on screening for common chronic diseases rather than newborn screening. These principles guide policy decisions regarding appropriate screening targets, based on factors such as the feasibility of early detection and the availability of an acceptable treatment. Wilson and Jungner also described the practices essential to operationalize screening, including data collection and analysis, provider education and community engagement. The criteria were updated in 2008 by Andermann et al. to reflect evolving societal and other influences with a focus on equity, autonomy, and quality assurance. Specifically, the revised framework includes a new criterion that ‘the programme should promote equity and access to screening for the entire target population.’ The Wilson and Jungner principles are not being widely utilized in current DNA-based population screening in the United States. Revisiting these criteria would be important in order for DNA-based population screening programs to reach their potential.

Current local and statewide DNA-based population screening efforts are being led by academic institutions and regional health systems. These programs could be enhanced by a national advisory committee with recommendations such as exists with NBS. State NBS systems evolved independently for more than 30 years before resulting disparities led to national calls for standardization. In response, the U.S. Department of Health and Human Services (HHS), Health Resources and Services Administration commissioned then American College of Medical Genetics to outline a process (Watson et al., 2006) for guidance to align and support efforts nationally. Primary outcomes were the development of the Recommended Uniform Screening Panel (RUSP) in 2002 and the establishment of the Advisory Committee on Heritable Disorders in Newborns and Children (ACHDNC) in 2003. ACHDNC membership is professionally diverse, drawing from public health NBS systems, clinical experts, rare disease advocates, and federal regulatory and service agencies. The Committee advises the HHS Secretary on NBS system priorities and needs, applying a decision matrix aligned with the Wilson and Jungner framework to examine and prioritize conditions for universal screening. The Committee has recently recognized that various factors, including the lasting impacts of structural racism, demand increased attention and commitment to achieve equitable outcomes. These practices are crucial to maintaining the wide public support and success of NBS as a public health practice. Developing and applying similar frameworks to newer DNA-based population screening practices is imperative to avoid increasing existing disparities surrounding health outcomes for a growing number of treatable conditions. Without similar frameworks the implementation of DNA-based population screening has been haphazard, dependent upon the buy-in of leaders at various institutions and hospitals, technology-led, and consumer-driven. While these programs are not restricted by the payer issues discussed above, they depend on funding from partners (e.g., pharmaceutical companies, state and federal research funds) which can introduce financial drivers that are incompatible with equitable recruitment. Many of these studies are incentivized to recruit as quickly as possible, regardless of the make-up of the cohort, resulting in inherent disparities in attempts at comprehensive and equitable integration.

## Other Public Health and Genetic Screening Frameworks to Consider in DNA-Based Population Screenings

Several current DNA-based population screening efforts utilize lists of genetic tests developed for other purposes such as the Tier 1 applications from the Centers for Disease Control & Prevention (CDC). The CDC has categorized genetic tests into tiers based on the evidence and/or consensus for their use in practice. Tier 1 applications are those having significant potential for positive impact on public health in specific settings. These applications are based on available evidence-based guidelines and recommendations (Bowen et al., 2012). There is currently no list of genetic tests or framework in DNA-based population genomics that integrates and/or prioritizes inequities in populations.

Additionally, there currently is no national public health genomics infrastructure for DNA-based based population screening in the United States. Current DNA-based population screening efforts at the state and local levels are occurring independently with finite funding from industry, foundations, governmental and research entities. Given this limited and uncertain funding, sustainability and time, DNA-based screening programs are focused on volume and speed at the expanse of equity. The Evaluation of Genomic Application in Practice (EGAPP™) was a previously funded effort by the CDC (Veenstra et al., 2012). EGAPP™ existed from 2005 to 2014 and provided a framework and national advisory role to select and evaluate genomic screening applications for specific clinical indications and populations. While there were shortcomings of this process, EGAPP served as a model for a federally funded entity which could partner with local and statewide DNA-based population screening programs and provided guidance about how to ensure equity across screening efforts. There is also a need for federal and state policies that support DNA-based population screening efforts and provide secure funding to ensure sustainability and health equity.

Furthermore, current DNA-based population screening efforts do not appear to use other key public health concepts, such as the Ten Essential Public Health Services. The Ten Essential Public Health Services was initially created in 1994 to provide a framework to describe the activities that public health systems should undertake in all communities (Castrucci, 2021). The framework was revised in 2020 with an explicit focus on equity to reflect public health values and social justice. More specifically, the visual representation of this framework places equity at the core. This is meant to be ‘a reminder of how public health must center on communities that have been historically marginalized in their work’ (Castrucci, 2021). DNA-based population screening needs to similarly place equity at the core of all activities. We would like to suggest creation of a new network of DNA-based population screening programs with national, state and local partners to share best practices and to collaborate on development of a framework that priorities health equity.

## Examples of DNA-Based Screening Efforts

Several institutions (Table 1) including Mayo, Geisinger, Intermountain Healthcare, and NorthShore University HealthSystem, have developed and implemented personalized medicine testing programs (Lemke et al., 2017; Schwartz et al., 2018; Pritchard et al., 2021). Northshore’s DNA-10K program specifically targeted the idea that scalable delivery of genomic medicine requires collaboration between genetics and non-genetics providers, implementing a combined primary care-genetics provider approach. Individuals who agreed to testing consented online in advance of their annual preventive care visit, at which time their primary care physician could place an order for clinical testing. The framework for NorthShore’s Personalized Medicine initiatives was developed at the local level *via* review and included assessment of CDC Tier 1 conditions and other guidelines, including the National Comprehensive Cancer Network (NCCN) Cancer Gene guidelines, American Heart Association (AHA)-supported cardiac genes, and ClinGen curated genes for disease association, as well as American College of Medical Genetics & Genomics (ACMG) incidental finding guidelines (a *de facto* guideline in the field of genomic population screening).

**TABLE 1:** Selected population genomic screening initiatives.

Project	Target population	Year initiated	Testing and return of results	Findings
Ohio Colorectal Cancer Prevention Initiative^a^	Ohio residents	2013	3,310 colorectal cancer patients (CRC) underwent universal tumor screening (UTS) for mismatch repair (MMR) deficiency	Approximately 16% of patients had MMR deficiency. Pathogenic germline variants in cancer susceptibility genes were found in 234 patients, representing 7.1% of the entire cohort and 16% of the 1,462 patients who received MGPT. Pearlman et al. (2021)
			Germline multigene panel testing (MGPT) was performed for patients with MMR deficiency	
Renown Healthy Nevada (with 23 and Me, Helix)^b^	Nevada residents	2016	>26,906 individuals from throughout the state of Nevada assessed for ancestry, LS, hereditary breast and ovarian cancer syndrome (HBOC), and familial hypercholesterolemia (FH)	1.33% (1 in 75) individuals had one of these three conditions. Among them, only 21.9% had clinically relevant disease, 25.2% had a family history of a relevant disease, and 90% had not been previously diagnosed. Grzymski et al. (2020)
		2018		
Geisinger MyCode (with Regeneron Pharmaceuticals)^c^	Geisinger patients	2014	>142,000 participants had their data analyzed for actionable hereditary disorders. (MyCode Scorecard, April 2022)	Almost 3,400 participants have received results to date, 48.1% with LS, HBOC or FH diagnoses. (MyCode Results Reported, April 2022) Studies on this cohort revealed that 87% of 351 individuals with LS, HBOC, and FH diagnoses were unaware of their genetic status before testing and 84% were eligible for additional interventions to mitigate disease risk. Buchanan et al. (2020)
NorthShore DNA-10K (with Color)^d^	NorthShore patients	2019	10,000 participants and provided patients with results for 60 genes associated with hereditary cancer and cardiac conditions, a 14-gene panel for pharmacogenomics (PGx) testing, ancestry and common trait information (such as lactose intolerance)	99% of eligible physicians ordered testing for a patient and more than half said DNA-10K has already provided a direct clinical benefit to patients. Nearly 80% of participants consented to participate in third party research and 70% said that the program “enabled them to better manage their personal health”. (Northshore Press Release)
Mayo Clinic Tapestry study (with Helix)^e^ ^,^ ^f^	Mayo Clinic patients	2020	Returning ancestry results and actionable genetic findings derived from whole exome sequencing (WES) testing, starting with LS, HBOC, and FH.	Results pending
Intermountain HerediGene: Population Study (with deCODE Genetics/Amgen)^g^	Utah and Idaho residents	2019	Return of results planned	Results pending

a Cancer.osu.edu/our-impact/community-outreach-and-engagement/statewide-initiatives/statewide-colon-cancer-initiative.

b Healthynv.org/.

c Geisinger.org/precision-health/mycode.

d Northshore.org/personalized-medicine/our-services/color-genetics-test/.

e Genomeweb.com/genetic-research/regeneron-mayo-ink-pact-sequence-genotype-100k-patient-samples.

f Mayo.edu/research/centers-programs/center-individualized-medicine/research/clinical-studies/tapestry.

g Intermountainhealthcare.org/heredigene.

Two examples of state level DNA-based population screening strategies exist in Ohio and Nevada. Ohio leverages universal tumor screening with germline multigene panel testing for Lynch syndrome (LS) among all colorectal cancer patients. Nevada uses population screening in the general public for three Tier 1 CDC applications that have been defined in more narrowly defined populations. The Ohio study provides an important example of centralized expertise that could be utilized by other DNA-based population screening efforts at the state and local levels. The study also demonstrates that DNA-based population screening efforts with germline multigene panel testing (MGPT) will detect more patients and that wide-spread screening efforts involving multiple health systems at the state level is feasible. The Healthy Nevada study provides support that DNA-based population screening efforts at the state level detect previously undiagnosed hereditary conditions with actionable prevention measures.

These programs have demonstrated significant success at recruiting participants, returning actionable genetic results at scale, and engaging local researchers and physicians to participate in the programs. However, they have been critiqued for a lack of racial and ethnic diversity. The races and ethnicities of the participants are often similar to the population served but favor white, non-Hispanic enrollment. Buchanan et al. (2020) reports that 96.1% of MyCode participants are white and 97.5% are non-Hispanic/non-Latino, compared to 93.2 and 96.0% of active Geisinger patients respectively. Grzymski et al. (2020) reported similar consistency between the racial and ethnic makeup of the Healthy Nevada cohort (81% white, 10% Hispanic/Latino, 3% Asian, 1% African American compared to the Renown Health System (72, 10, 3, 3%, respectively), but an oversampling of white participants and underrepresentation of racial and ethnic minorities compared to Washoe County (63, 25, 5, 2% respectively).

It is critical that this history not be established as the norm for population genomic studies. As recently highlighted by the *All of Us* Research Initiative, oversampling of racial and ethnic minorities and other marginalized groups is achievable with targeted and purposeful effort. Currently, >50% of the *All of Us* cohort identifies as a racial/ethnic minority and >80% are traditionally underrepresented in biomedical research based on gender identity, sexual preference, age, disability status, etc. (https://www.researchallofus.org/data-tools/data-snapshots/) In March 2022, *All of Us* announced the release of nearly 100,000 whole genome sequences from this population, demonstrating the ability to recruit diverse participants for population genomic sequencing efforts specifically. (https://allofus.nih.gov/news-events/announcements/program-releases-first-genomic-dataset).

Elyse Azriel, Lynch syndrome previvor, captures the success of such local efforts and the promise of DNA-based population screening:

“As a healthy and active 26-year-old, I had no idea that I might have an underlying genetic condition. During an annual physical, my doctor told me about a partnership that our hospital system, located in the northern suburbs of Chicago, had with a genetic testing company. The initiative called ‘DNA 10K’ was a population health program with the goal to enroll 10,000 patients for genetic testing. She encouraged me to enroll due to my dad's history of colon cancer at age 48. At first, I was hesitant to participate because I had just received a negative result on a direct-to-consumer test six months prior. However, when my doctor explained that this genetic test was more comprehensive and could potentially detect a variant that was more relevant to my family history of colon cancer, I decided to go ahead with it.

This is when I first heard the words “Lynch Syndrome”. I found out that I am positive for this genetic variant, which is likely pathogenic and means that I have a higher likelihood of developing several different types of cancers including colorectal and endometrial cancer. Luckily, I am a previvor, which means that I found out that I have Lynch Syndrome prior to ever developing cancer. I also have the privilege of accessing healthcare providers and resources such as colonoscopies and uterine biopsies annually to monitor for any new cancer. Three years later, I am relieved that I am still cancer free”

## Discussion

DNA-based population screening has the promise to improve health of all people in all communities. However, if current efforts continue without clear principles and frameworks, there will be continued harm and health inequities especially for those populations in greatest need. There is an urgent need for ‘A Call to Action’ that keeps equity at the core and involves all partners in DNA-based population screening efforts. Precision public health can use the framework of past and current initiatives in newborn screening as a basis to expand access and equity of DNA-based population screening. Potential actions to consider are:

• Identification and dismantling of systemic barriers that result in health inequities of genomic screening efforts to assure equitable access for people in all communities
• Creation of a national multidisciplinary advisory committee with representation from multiple underserved populations to improve current and inform future DNA-based population screening efforts
• Utilization of well-described framework(s) and criteria such as the revised Wilson and Jungner to guide screening decisions about appropriate conditions to include in DNA-based population screening initiatives
• Adoption of the newly updated Ten Essential Public Health Services with the core of equity in all efforts at the local, state, and national levels

We must embrace the wisdom of Candace Henley to “work hard to take care of the neediest members of our community and provide them with unconditional supportˮ.

## Data Availability

The original contributions presented in the study are included in the article, further inquiries can be directed to the corresponding author.
